# Vitamin A, systemic T-cells, and the eye: Focus on degenerative retinal disease

**DOI:** 10.3389/fnut.2022.914457

**Published:** 2022-07-18

**Authors:** Arun J. Thirunavukarasu, A. Catharine Ross, Rose M. Gilbert

**Affiliations:** ^1^Corpus Christi College, University of Cambridge, Cambridge, United Kingdom; ^2^University of Cambridge School of Clinical Medicine, Cambridge, United Kingdom; ^3^Department of Nutritional Sciences, The Pennsylvania State University, University Park, PA, United States; ^4^Cambridge University Hospitals NHS Foundation Trust, Cambridge, United Kingdom; ^5^NIHR Biomedical Research Centre at Moorfields Eye Hospital NHS Foundation Trust and UCL Institute of Ophthalmology, London, United Kingdom

**Keywords:** vitamin A, retina, gut microbiome, T cell, Stargardt’s disease, age - related macular degeneration, carotenoids, retinoids

## Abstract

The first discovered vitamin, vitamin A, exists in a range of forms, primarily retinoids and provitamin carotenoids. The bioactive forms of vitamin A, retinol and retinoic acid, have many critical functions in body systems including the eye and immune system. Vitamin A deficiency is associated with dysfunctional immunity, and presents clinically as a characteristic ocular syndrome, xerophthalmia. The immune functions of vitamin A extend to the gut, where microbiome interactions and nutritional retinoids and carotenoids contribute to the balance of T cell differentiation, thereby determining immune status and contributing to inflammatory disease around the whole body. In the eye, degenerative conditions affecting the retina and uvea are influenced by vitamin A. Stargardt’s disease (STGD1; MIM 248200) is characterised by bisretinoid deposits such as lipofuscin, produced by retinal photoreceptors as they use and recycle a vitamin A-derived chromophore. Age-related macular degeneration features comparable retinal deposits, such as drusen featuring lipofuscin accumulation; and is characterised by parainflammatory processes. We hypothesise that local parainflammatory processes secondary to lipofuscin deposition in the retina are mediated by T cells interacting with dietary vitamin A derivatives and the gut microbiome, and outline the current evidence for this. No cures exist for Stargardt’s or age-related macular degeneration, but many vitamin A-based therapeutic approaches have been or are being trialled. The relationship between vitamin A’s functions in systemic immunology and the eye could be further exploited, and further research may seek to leverage the interactions of the gut-eye immunological axis.

## Introduction

**FIGURE 1:**
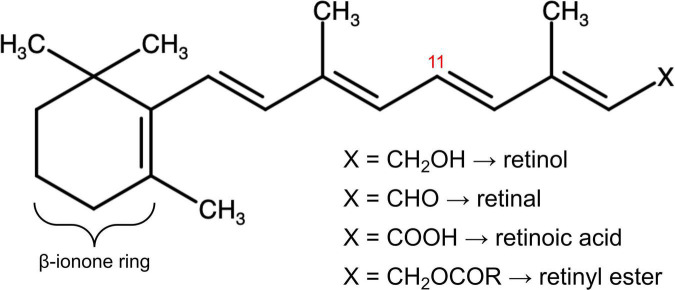
The molecular structure of vitamin A in its multiple forms. The chemical group denoted X defines retinol, retinal, retinoic acid, and retinyl esters depending on its composition. Stereoisomeric 11-cis and 11-trans retinoids depend on the three-dimensional arrangement of the vitamin A molecule around the double bond at the eleventh carbon residue, labelled in red. The ß-ionone ring, modified in synthetic forms of vitamin A, is also labelled.

The first discovered vitamin, known initially as “fat-soluble A” (1, 2), and later as vitamin A, is comprised of fat-soluble organic compounds, or “vitamers,” chemically related to vitamin A1, retinol (3, 4). Although much was learned about the nutritional activity of this essential component of the diet in the first two decades following its discovery, it was not until the 1930’s that the chemical structure was elucidated by Karrer, who was awarded a Novel Prize for this and other discoveries (5). The chemical name (IUPAC nomenclature) of retinol is (2*E*,4*E*,6*E*,8*E*)-3,7-dimethyl-9-(2,6,6-trimethylcyclohexen-1-yl)nona-2,4,6,8-tetrae n-1-ol. The structure (Figure 1) contains an unsaturated ring more commonly referred to as a β-ionone ring, a methyl substituted unsaturated side chain, and a terminal alcohol moiety. This hydroxyl group is the site of enzyme-catalyzed oxidation reactions that yield retinal and, subsequently, retinoic acid (RA), considered the two major forms of vitamin A in terms of physiological activity. Vitamin A deficiency manifests as a characteristic clinical syndrome, and an intake that is either too high or low can have a wide range of pathological effects.

Retinoic acid functions as a ligand for nuclear RA receptors (RARs), which are among the most studied of nuclear hormone receptors, mediating both the organismal and cellular effects of intracellular retinoic acids and their synthetic analogues (6). The RAR family has three main isoforms: α, β, and γ, capable of forming heterodimers with retinoid X receptors (RXRs). RAR-RXR heterodimers interact with retinoic acid response elements (RAREs) within the promoters of RA-responsive genes (6, 7). Retinoids regulate many essential biological processes, acting as potent gene expression modulators during embryogenesis, organogenesis, cell growth, differentiation and apoptosis, including the embryonic development and normal metabolism of the eye (6, 8).

**FIGURE 2:**
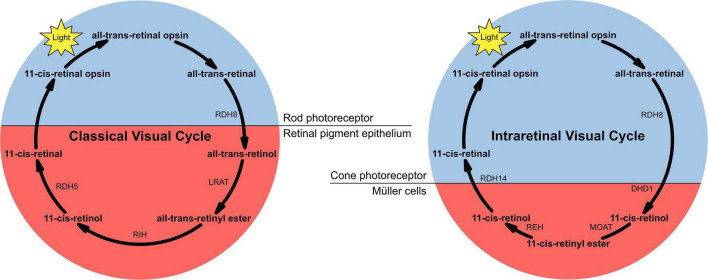
The two versions of the visual cycle, classical and intraretinal, a series of chemical reactions, mostly enzyme-catalysed, underlying chromophore regeneration in rods and cones, respectively. Intra- and extra-photoreceptor processes are shaded blue and red, respectively. Rods work with the retinal pigmented epithelium in the classical visual cycle, whereas cones work with Müller cells in the intraretinal visual cycle.

The neurosensory retina is a layered tissue which lines the back of the eye, communicating with the brain via the optic nerve. Blood is supplied to the neurosensory retina by retinal blood vessels originating from the central retinal artery. Transport across retinal blood vessels is limited by endothelial tight junctions, which constitute the inner blood–retinal barrier. The optic nerve is comprised of ganglion cell axons, the “output neurons” of the retina. The retina is built up of three layers of cell bodies and two plexiform layers of synapses, clearly defined on histological sections. The innermost layer is the ganglion cell layer. The inner nuclear layer, featuring the cell bodies of bipolar, amacrine, and horizontal cells, is sandwiched by an internal plexiform layer and ganglion cell layer on one side, and outer plexiform layer and outer nuclear layer on the other. The outer nuclear layer features the cell bodies of most of the photoreceptors: the rods and cones. A third class of photoreceptor, intrinsically photoreceptive ganglion cells, is situated in the ganglion cell layer (9, 10). All photoreceptors depend on a vitamin A-derived chromophore to detect light (11). The chromophore associated with light transduction proteins, opsins, and undergoes photoisomerisation during transduction. Expended chromophore then enters the visual cycle, a sequence of reactions which facilitates regeneration and re-association with opsins for further transduction (Figure 2). While in rods, the visual cycle involves the rod itself and retinal pigment epithelium (RPE) overlying the neurosensory retina, cones operate with Müller cells instead, a sort of glial support cell in the retina (12). Accumulation of retinoid products of these photoisomerisation and visual cycle processes underlies the pathophysiology of Stargardt’s disease (STGD), and similar products are associated with age-related macular degeneration (AMD).

T-lymphocytes (T-cells) are a main cell type of the adaptive immune system and circulate in the peripheral blood, constituting approximately 80% of the total lymphocyte population (13). They express a T-cell receptor (TCR), which interacts with peptides complexed with Major Histocompatibility Complex (MHC) antigens (14) and has the capacity to “recognise” diverse antigens from the host body, pathogens, tumours, and the environment. In addition to facilitating adaptive immune responses, T cells are implicated in many inflammatory and autoimmune diseases (15, 16). Various subsets of T-cell are defined by the cell surface markers, cytokines, and transcription factors that they express. The T-helper 17 (Th17) cell subset is characterised by two master regulator orphan nuclear receptors: retinoid orphan receptor (ROR) γt and RORα, named for their homology to the nuclear retinoid receptor proteins; and by the expression of the RORγt transcription factor and its signature cytokine, IL-17 (17–20). The Th17 lineage, in addition to the Th1 subset (characterised by expression of the T-bet transcription factor), is implicated in chronic inflammatory and autoimmune disease. There is evidence that both of these Th subsets may act as effector cells in the pathogenesis of ocular autoimmunity (21, 22). T-regulatory (Treg) cells function as a natural mechanism to suppress autoreactive T-cells and are characterised by expression of CD4 and CD25 surface receptors, a forkhead box P3 (FoxP3) transcription regulator and production of cytokines such as IL-10 and transforming growth factor β (TGF-β) (21, 23, 24). The bioactive metabolite of vitamin A, RA, has been shown to oppose Th17 cell commitment (19) and, as discussed further below, is capable of inducing Treg that are critical for maintaining immune homeostasis and for opposing the induction of autoimmune T cells (20).

Fully differentiated T cells have the capacity to modify their gene expression to adapt to new conditions. Differentiation of naïve CD4+ cells into T-cell subsets such as effector Th1 and Th17, or induced Treg (iTreg) cells may depend on epigenetic regulation in response to changing environmental conditions, in addition to determination by cell fate programmes (25, 26). Therefore, the ability of the local environment to provide an adequate supply of RA to promote iTreg maintenance, or to be conducive to the conversion of Th17 cells into iTreg cells, is important (20). This is especially significant within the mucosal environment of the intestine, where dietary nutrients such as vitamin A and the bacteria of the microbiome act to co-regulate the balance of pro-inflammatory and anti-inflammatory T cells (27, 28). There are likely a myriad of interactions between the host and microbiome that shape the immune profile of an individual at any one time, and the gut microbiome is specifically implicated in ocular inflammatory diseases, including AMD (29, 30).

In this review, vitamin A physiology is described, including intake, absorption, and delivery to dependent tissues; ocular usage of vitamin A, with an emphasis on retinal phototransduction and the visual cycle; as well as the role of vitamin A in T-cell mediated inflammation. The gut-eye axis is discussed, and its reliance on normal vitamin A metabolism and T cell function is explored. The pathophysiology of STGD (STGD1; MIM 248200) and AMD are then outlined, as diseases associated with chronic inflammatory and local parainflammatory processes, driven by aberrant generation of vitamin A derivatives, and/or retinoid-related disturbances in T-cell homeostasis in the microbiome, influenced by dietary vitamin A. Disruption in the interrelationships between nutrition, the microbiome, and host metabolism may underlie degenerative retinal diseases like STGD and AMD, presenting interesting therapeutic targets. Potential retinoid-based treatments for STGD and AMD are discussed, some of which have been and are currently being trialled in human patients. Potential future directions for research are posited, based on this evidenced vitamin A-related pathophysiology and therapy. In particular, a nutritional approach to improving outcomes is considered, with an overview of the complexity associated with dealing with patients exhibiting different phenotypes, and at different disease stages.

## Nutritional forms of vitamin A and their sources

Forms of vitamin A present in the diet include preformed retinol, contained in foods of animals origin and mainly in the form of retinyl esters, and the provitamin A carotenoids such as β-carotene. *De novo* synthesis of carotenoids is restricted to plants and microorganisms, meaning that animals like humans must consume either preformed vitamin A, obtained in foods of animal origin, or provitamin compounds (5). Important dietary sources of preformed vitamin A, are found in the highest concentrations in avian and mammalian liver, instant powdered breakfast drinks, ready-to-eat cereals, and margarine (31). The provitamin carotenoids that can give rise to retinol through metabolism include β-carotene, α-carotene, γ-carotene, and β-cryptoxanthin (31, 32), which are found in highest concentrations in green leafy and yellow vegetables (33). Several other dietary carotenoids, such as the xanthophylls zeathanthin, lutein, and lycopene, are absorbed into plasma and exert protective antioxidant activities; however, these xanthophylls are not precursors of retinol or its metabolites. As described further below, retinol is metabolized to two bioactive compounds, retinal, the form of the vitamin that is essential for vision, and RA, which is essential as a signalling ligand for nuclear retinoic acid receptors, RARs.

### Nutritional supplements

Vitamin A present in nutritional supplements is generally of manufactured origin, produced in the form of retinyl palmitate or retinyl acetate for greater stability. When vitamin A is given as a supplement to young children 6 months–5 years of age, as recommended by WHO in low- and middle-income countries in which deficiency is a public health problem, the form is most often retinyl palmitate, provided as a high-dose capsule once every 4–6 months (34).

### Non-nutritional retinoids

In addition to the naturally occurring forms above, many synthetic vitamin A derivatives, referred to as retinoids (35), have been synthesized and tested for their potential medicinal value. These can be classified in generations, with the first generation being natural compounds listed above including the acidic acid form RA; second generation compounds derived from the first generation, such as acitretin (36), which generally have a modified ring structure in place of the β-ionone ring; a third generation derived from the second, such as bexarotene (37); and recently a single fourth generation compound, trifarotene (38). Several of these retinoids are used in clinical contexts, including in the treatment of certain skin diseases (39) and cancers (40).

## Metabolism of dietary vitamin A

**FIGURE 3:**
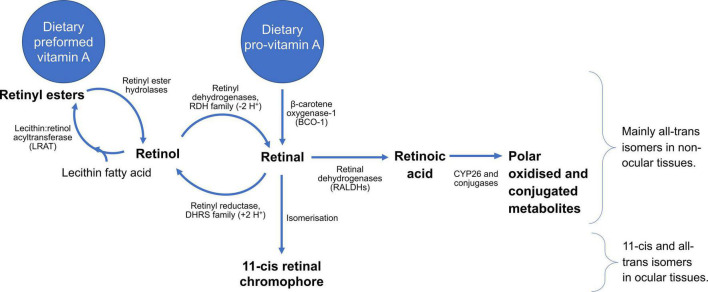
Summary of the metabolism of vitamin A, running from different dietary sources through generation of bioactive metabolites, as well as reactions required for clearance. All-trans stereoisomers predominate away from the eye, whereas the eye, and particularly the retina, feature 11-cis and all-trans isomers, crucial for vision.

Vitamin A metabolism comprises a complex series of reactions which include the cleavage of carotenoids, the esterification of retinol and hydrolysis of retinyl esters, a series of redox reactions that interconvert retinol and retinal in a reversible manner, and a terminal oxidation reaction which coverts retinal into retinoic acid. Additional enzymatic conjugation reactions serve to produce water-miscible forms of vitamin A for excretion. The enzymes principally responsible for these interconversions are indicated in Figure 3. Notably, retinol and retinal can cycle between these forms, while esterification of retinol provides a storage form of the vitamin, often the most abundant form in most tissues, which can then be drawn upon through retinyl ester hydrolysis to produce retinol, as required. Carotenoids, retinyl esters, and retinol itself can be considered as precursors for the two principal bioactive forms, retinal and RA. Both forms are critical for the health of the eye, therefore dietary vitamin A can be traced from uptake to its delivery to the eye, in addition to the reactions that take place in the cells of the retina and cornea.

A vast array of proteins are involved in the processes underlying absorption, such as for carotenoid cleavage (5, 41, 42), retinyl ester hydrolysis (43–45), facilitated diffusion into cells (46–48), intracellular trafficking of retinol in the enterocytes (31), re-esterification of retinoids (49, 50), and sequestering of free retinol (51). Mutations in the genes encoding these proteins may affect vitamin A absorption (52–54), and potentially lead to vitamin A deficiency syndromes, as discussed below (55).

### Intestinal processing

In the lumen, dietary vitamin A, mostly as retinol esters, must be solubilised with bile salts and products of lipid digestion to form mixed micelles, then, once the retinyl esters are in micellar form, they can be hydrolysed by pancreatic lipases to liberate retinol, which is then absorbed from the micelle across the enterocyte’s brush border and into the cytosol. β-carotene also requires micellisation for absorption, but the carotene molecule enters the enterocyte intact through the brush border transmembrane transporter ABCA1 (31). Inside the cell, most of the newly absorbed β-carotene is cleaved at the central 15,15′ double bond by the soluble enzyme β-carotene monooxygenase-I (BCO-1); this cleavage immediately produces two molecules of retinal that can then be reduced by retinal reductase to form retinol. β-Carotene may also undergo eccentric cleavage, in which case at most one molecule of retinal is formed, however, this route is considered secondary (56). Moreover, small amounts of retinoic acid are generated during the absorption of vitamin A and this metabolite may play a regulatory role in the intestine or be transported from the intestine via the portal vein (57).

As retinol is insoluble in the aqueous cytosol, its intracellular trafficking and further metabolism require that it be bound to a cellular retinol-binding protein (CRBP). In enterocytes the predominant form of CRBP is CRBP-II, which binds retinol that is derived either from metabolism of preformed vitamin A or carotenoids, and chaperones it to the esterifying enzyme lecithin:retinol acyltransferase (LRAT), which produces newly esterified retinol (58). Although the absorptive pathways for retinol and β-carotene converge at the CRBP-II–LRAT step, there are some differences in overall efficiency. For preformed vitamin A, the efficiency of absorption is typically around 70%, whereas for carotenoids, it is typically 20–30%, being lower as the ingested dose increases (5, 59). A feedback mechanism has recently been described whereby retinoic acid acting as a ligand for RAR induces an inhibitory transcription factor, ISX, that blocks the transcription of the ABCA1 and BCO-1 required for carotene uptake and cleavage, and subsequent retinol esterification (60).

Moreover, in humans and a few other mammals, a small fraction of β-carotene escapes cleavage, becoming incorporated directly into the lipid core of nascent chylomicrons. In this manner, the chylomicrons, which are present in plasma only in the postprandial state, transport not only triglycerides and other newly absorbed dietary lipids, but small quantities of fat-soluble vitamins including retinyl esters and carotene, the quantity of which depends on the amounts in the recently ingested meal (5). β-Carotene can be transformed sequentially to provide the photoreceptor pigments, as can preformed vitamin A (61).

After the newly formed chylomicrons enter the circulation, their triglycerides are rapidly hydrolysed by lipoprotein lipase to provide fatty acids to tissues, leaving in the circulation the so-called chylomicron remnants that are taken up by numerous tissues, with most entering the hepatocyte through lipoprotein receptors. Once retinol has entered the liver, after further metabolic processing (5), retinol can either be stored in hepatic stellate cells as retinyl esters, mediated by CRBP (type 1) and LRAT, or it can be secreted into plasma bound to its transport protein, retinol-binding protein (62). Esterification predominates under conditions of adequate vitamin A intake, and significant vitamin A stores may build up in the hepatic stellate cells over time. When, however, tissues are depleted of vitamin A, the newly absorbed retinyl esters in the chylomicron remnants are hydrolysed and the retinol is shunted towards the holo-RBP secretory pathway and into plasma (5).

### Plasma transport

When vitamin A is required by tissues, the hepatic retinyl ester pool in the hepatic stellate cells is drawn upon by hydrolysis reactions, and the retinol is transferred back to the hepatocytes where newly synthesised retinol-binding protein (RBP) binds the retinol and the complex, known as holo-RBP, is secreted into plasma. Holo-RBP consists of one molecule of all-trans-retinol per molecule of RBP, which then associates in plasma with a co-transport protein, transthyretin (63). In the fasting state and, in fact, during most of the day except in the postprandial period after consumption of vitamin A-containing meals, the majority of plasma vitamin A is in the form of holo-RBP (5). In healthy humans, holo-RBP concentration is quite steady over time, between 1 and 3 μmol/L, and is regulated in this relatively narrow range over a much wider range of liver vitamin A concentrations (64). Because RBP synthesis is essential for the secretion of holo-RBP, it has been of interest to understand conditions that may compromise the synthesis and secretion of holo-RBP and thereby result in low serum retinol and RBP concentrations. Conditions that limit holo-RBP production include inadequate protein or energy consumption, and zinc deficiency, and renal diseases leading to hyperfiltration or reduced reuptake in the proximal tubules. Additionally, both experimental and clinical data have shown that circulating levels of holo-RBP, retinoids, and carotenoids are low during infection or inflammation, independently of vitamin A nutritional status (65), and may affect the response to vitamin A supplementation (66). This is due in part to RBP’s nature as a negative acute phase protein; its concentration falls rapidly in response to inflammatory stimuli (67). Thus, low holo-RBP levels in patients with inflammatory conditions should be interpreted with caution, as the underlying cause could be nutritional or it could be due to inflammation itself (63).

## Vitamin A and the eye

### Retinol in the retina

The retina must acquire retinol from holo-RBP circulating through the capillaries in the choroid. A receptor for RBP, Stra6, was described, cloned and shown to be most abundantly present in the basolateral plasma membrane of the retinal pigmented epithelial (RPE) cells (68). Stra6 binds RBP and allows for the uptake of retinol, which very likely is immediately picked up by CRBP-1 on the cytosolic side and, then, similar to other tissues, esterified by LRAT (69). Recent studies have identified that Stra6 transports retinol both into and out of cells, with calmodulin as an interacting regulatory protein (70).

The retina is unique in having an abundance of 11-cis-retinoids, the essential chromophore for all photoreceptor cells; vitamin A is thus an absolutely required nutrient for vision (71). The RPE cells contain retinyl esters in both the all-trans- and the 11-cis configuration, suggesting that LRAT also esterifies 11-cis-retinol produced by isomerisation in the retina. The concentration of vitamin A is the highest in the eye of any tissue, reaching as high as 3 millimolar in the retina (72, 73), mostly in the form of 11-cis-retinal bound in a Schiff base with rhodopsin in rods or photopsins in cones. Opsins are G-protein coupled receptors (GPCRs), a class of protein which underlies vision in all animals, including in man (11). In addition to rhodopsin in rods for vision in scotopic-mesopic light intensities, and three photopsins in cones for colour-tuned vision in mesopic-photopic light intensities, humans express a fifth opsin, melanopsin, which facilitates non-image forming vision (10, 74–77). The molecular interaction between the specific GPCR and chromophore tunes light sensitivity to different wavelengths (78, 79). Each of the five human opsins has a different and characteristic absorption spectrum due to their slightly different interactions with the 11-cis-retinal chromophore; dependent on a relatively small number of key amino acid residues (80, 81). Long, medium, and short-wavelength sensitive photopsins underlie colour vision by facilitating visual contrast owing to preferential responses to red, green, and blue light, respectively (77, 79). At lower light intensities, rods facilitate vision with rhodopsin-dependent transduction, due to both specific photoreceptor characteristics and downstream signalling pathways adapted to optimise sensitivity (82, 83). The human retina is thus described as “duplex,” as it depends on two photoreceptor classes at different ambient light levels, developed in discrete evolutionary steps (84).

At the molecular level, a photon of light provides the energy to switch the chromophore’s structure, from 11-cis to all-trans retinol. This increases the physical strain on the molecule, likened to a spring, with relief on tension conferred by altering the conformation of the opsin, which thus activates a signalling cascade (85). As the photoisomerisation transduction process results, at least transiently, in the formation of “free” retinoids, and as aldehydes in particular are highly reactive molecules, especially in the presence of amines, researchers have looked for and identified a number of adduct products of retinal and cellular amines. Those identified include A2E, a bisretinoid condensation product of retinal and phosphatidyl ethanolamine (PE), its isomer iso-A2E, and other products such as lipofuscin (86). These by-products of the photoreceptor processes are difficult for cells to clear and, though reversible, may accumulate. A2E, lipofuscin, and other bisretinoids are discussed below as the metabolites responsible for the pathogenesis of Stargardt’s disease.

### Corneal vitamin A dependence

The cornea is essentially an avascular tissue, yet it also requires retinol for the normal structure and function of the conjunctival and corneal epithelial cells. The synthesis of RBP by the lacrimal gland and the presence of RBP in tear fluid was noted some time ago (87, 88). While relatively little is understood of the mechanisms by which RBP enters the lacrimal glands and is taken up by corneal cells from tear fluid, it seems likely that tears may play an important role in providing retinol to this epithelium. The lacrimal gland, cornea and its cells contain retinoid receptors and respond to all-trans-retinoic acid (89–91). Conjunctival impression cytology, in which cells lifted from the outer edge of the conjunctiva are stained for mucins, has been used as a clinical tool for assessing vitamin A deficiency (92); effective as vitamin A deficiency presents clinically as corneal xerosis, colloquially called “dry eye.” The broader ocular syndrome associated with vitamin A deficiency, xerophthalmia, which involves the cornea and retina, is described in greater depth below.

## Vitamin A’s influence on T cell function

Shortly after vitamin A was initially identified in the early 1900s, it was termed “the anti-infective vitamin” (93). It has important roles in innate immunity with regards to barrier (*i.e.*, skin), neutrophil, macrophage, and NK cell function (93). Furthermore, it is thought be a key player in immune regulation through its effects on adaptive immunity, particularly via T-helper (Th) cells (7, 93). Vitamin A mediates these effects directly in the bioactive form of RA, and, also, via vitamin D and the vitamin D receptor (VDR). To mediate an anti-inflammatory effect, active vitamin D, calcitriol binds to the VDR to form a VDR-D complex which then binds to the RXR receptor, activated by its ligand RA. It is this heterodimer complex, RXR–RA/VDR-D, dependent on vitamin D and vitamin A, that controls the expression of several genes involved in inflammatory and autoimmune processes in chronic diseases by inhibiting the pro-inflammatory transcription factor NF-κB and down-regulating the synthesis of inflammatory molecules (7, 94, 95). It is also worth noting that, given their common RXR nuclear binding partners, calcitriol and retinoic acid may antagonize each other’s effects (7, 96).

Retinoic acid is a critical signalling factor which drives differentiation of iTreg cells (20, 97–100). iTreg differentiation is favoured, for example, in the presence of TGF-β and adequate amounts of RA, but low IL-6 (20). RA indirectly promotes TGF-β-mediated Treg cell conversion by suppressing pro-inflammatory cytokine production, thereby stabilising FoxP3 expression. Generation of iTreg cells also occurs through direct binding of FoxP3 to RORα and RORγt, inhibiting their transcriptional activity (101, 102). RA produced by specific dendritic cell (DC) subsets, directly and indirectly modulates FoxP3 expression and increases the activation of extracellular-related kinase (ERK) signalling to promote FoxP3 expression (100, 103, 104). RA increases demethylation and histone acetylation of the FoxP3 gene locus and promotor, respectively (100). RA attenuates the deceleration of FoxP3 expression during Treg cell expansion and in inflammatory conditions, with superior efficacy compared with rapamycin, an mTORC1 inhibitor known to stabilise FoxP3 expression (100). RA also suppresses Th17 cell development by increasing TGF-β signalling and reducing the level of expression of the IL-6 receptor (105, 106). It is thought that when conditions favour immune tolerance, there is a balance between effector T cells and Treg cells, whereas imbalances occur in autoimmunity, which could be due to inadequate numbers of Treg cells, defects in Treg function or phenotype or reduced responsiveness of effector T cells to Treg-mediated suppression (107). Therefore relative increase in iTreg and the inhibition of Th17 development mediated by RA, can shift the balance of these cells toward more effective immune homeostasis (20).

## The T cell-mediated gut-eye axis

It is well recognised that the human immune system has a highly co-evolved relationship with the microbes that inhabit the human intestine, or microbiome, resulting in the co-maintenance of homeostasis between the host and resident microbes (108). This relationship is shaped throughout development and into adulthood, and contributes to the function of the gastrointestinal immune system, as well as playing an important role in health and disease throughout life (108). Dysbiosis is defined as disruption to the microbiome’s homeostasis, caused by imbalanced microbiota structure or function, and is an emerging feature of many non-communicable inflammatory diseases (109). The gut microbiome composition varies according to macro/micronutrient dietary habits (110–115). Different bacterial taxa modulate immune functionality, driving pro or anti-inflammatory activity (28, 116, 117). Thus, the composition of the microbiota community determines, in part, the level of resistance to infection and susceptibility to inflammatory diseases.

RA seems to play a predominant role in the homeostasis and homing of lymphoid populations of the gut-associated lymphoid tissue (GALT). It is synthesised in abundance by gut DCs (118, 119), induces the specific gut-homing molecules CCR9 and α4β7 integrin expressed by T cells, and also promotes GALT-related activity in B cells (7, 118). The balance between Th17 and iTreg cells and their ability to cross-regulate one another have emerged as crucial factors for normal mucosal immunity (19, 98, 101). The local microenvironment, which may include the cytokine milieu and the microbiome, has been shown to regulate the Treg/Th17 balance and influence cell plasticity; disruption of this balance may lead to the development of inflammatory disease (120–123). Paradoxical expression of effector CD4 T-cell transcription factors, such as T-bet and RORγt, by Treg has been suggested to enhance Treg suppressive capacity in murine models of inflammation (123–126). These murine models demonstrate that intestinal “double positive” RORγt^+^FoxP3^+^ Treg cells induced *in vivo* by the local microbiota display a stable suppressive phenotype and exist in dynamic balance with pathogenic Th17 (122, 125).

Induction of Treg cells in the small intestine appears to depend more on dietary antigens than microbes, according to experimental results in murine models. RA acts in concert with TGF-β for Treg cell generation (27, 119, 127), where abundant vitamin A from the diet is converted to RA by local epithelial cells and DCs (5, 128, 129). Moreover, RA induces the expression of CCR9 on Treg cells, which is required for their migration to small intestine (27, 127). RA-induced Treg cell differentiation underlies oral tolerance, as mice treated with a vitamin A-deficient diet display defects in the induction of oral tolerance (130). Thus, dietary antigens and RA-dependent Treg cells in the environment of the small intestine may modulate inflammation at distant sites immune-privileged sites, such as the retina. This is one potential mechanism of many by which the gut microbiome affects retinal health, potentially contributing to a wide range of chronic eye diseases, including AMD (29, 30).

Ocular immune privilege is a complex phenomenon that involves multiple components, starting with sequestration behind an efficient blood–retina barrier, through active local inhibition by soluble and surface-bound molecules that actively inhibit activation and function of adaptive and innate immune cells, and culminating in systemic regulation via induction of Treg (131). Failure of ocular immunological tolerance may lead to classical autoimmune-type inflammation, which may manifest in the eye as uveitis, an inflammatory disease of the choroid, which is often associated with inflammation of the overlying retina. It is hypothesised that RPE cells produce RA, thereby enabling bystander T cells to be converted into Tregs through TGFβ promotion, which can then participate in the establishment of immune tolerance in the eye (132). There is evidence that RORγt-expressing Th17 cells act as the effector cells in ocular inflammation (133), including B27^+^ anterior uveitis (17, 98, 101, 134). In a large clinical study of patients with non-infectious uveitis, it was shown that systemic Th17 cells were not associated with active ocular inflammation and that double positive FoxP3 + RORγt + Treg cells were not significantly associated with clinical remission of ocular inflammation (135). However, systemic Th17 cells from patients with active uveitic disease were observed to express FoxP3 during the disease resolution phase, in response to immunosuppressive treatment (135). There has been interest in glucocorticoid-resistant Th17 cells refractory to Treg suppression (136, 137), and these have been previously investigated in sight-threatening uveitis (138). Furthermore, a subset of “non-classic” Th17-derived Th1 have been described, which are hypomethylated at *RORC*, the gene encoding RORγ, and are thought to play a pivotal role in the establishment and persistence of inflammatory disease (139). These non-classic Th1 express CD161, a marker of steroid-resistant human Th17, induced by RORγ signalling (140). CpG methylation at *RORC* in systemic Treg appears to indicate clinical remission or clinical response to systemic corticosteroids in non-infectious sight-threatening uveitis (135), highlighting the potential role of RA in T-cell mediated ocular inflammation.

## Vitamin A deficiency and excess

Although the body stores vitamin A as retinyl ester in liver and to a lesser extent in adipose, lung, and other tissues, these stores may become depleted over time if the diet is consistently deficient in vitamin A, or stores may never develop in children who are not adequately breastfed and/or do not receive an adequate diet after weaning (141). Food deprivation, unbalanced diet, and malabsorption syndromes are the most common causes of deficiency (142–144). Worldwide, dietary deficiency is by far the most common cause, epidemic in developing countries and particularly affecting young children (145, 146). Dietary deficiency may also be observed in individuals with low general food intake, such as in anorexia or bulimia nervosa (142). Fat malabsorption syndromes can result in hypovitaminosis A, such as due to chronic pancreatitis, inflammatory bowel disease, and liver disease (142, 147).

Dietary advice regarding optimal vitamin A intake varies between jurisdictions, and is summarised in Table 1 (33, 148, 149). As discussed above, the many forms of vitamin A distribute differently across a wide range of foods. To deal with this variability, the vitamin A activity of biological substances and of the daily nutritional requirement may be expressed in terms of “retinol equivalents” (RE). 1 μg RE may be considered equivalent to 1 μg of retinol, 6 μg of β-carotene, or 12 μg of other provitamin A carotenoids (149). Daily intake for vitamin A can be met with any combination of preformed vitamin A or carotenoids that produces the correct quantity in RE terms.

**TABLE 1:** Summary of dietary advice regarding daily vitamin A intake from the United States of America (National Institutes of Health Office of Dietary Supplements, NIH ODS), United Kingdom (Scientific Advisory Committee on Nutrition, SACN), and European Union (European Food Safety Authority Panel on Dietetic Products, Nutrition, and Allergies, EFSA NDA).

Authority	Men	Women	Children
National Institutes of Health (33)	900 μg RE/day	700 μg RE/day; 770 μg RE/day if pregnant; 1,300 μg RE/day if lactating	400 μg RE/day at 0–6 months; 500 μg RE/day at 7–12 months; 300 μg RE/day at 1–3 years; 400 μg RE/day at 4–8 years; 600 μg RE/day at 9–13 years; adult values if over 14 years unless pregnant (750 μg RE/day) or lactating (1,200 μg RE/day)
Scientific Advisory Committee on Nutrition (148)	700 μg RE/day	600 μg RE/day; less than 3,000 μg RE/day if pregnant (supplementation not specifically advised)	350 μg RE/day at 0–12 months; 400 μg RE/day at 1–6 years; adult values for girls over 11 years and boys over 15 years
EFSA Panel on Dietetic Products, Nutrition, and Allergies [NDA] (149)	570 μg RE/day	490 μg RE/day; 700 μg RE/day if pregnant; 1,300 μg RE/day if lactating	190 μg RE/day at 7–11 months; 580 μg RE/day in boys aged 15–17 years; other figures derived from bodyweight

Vitamin A deficiency affects many physiological processes. The body’s epithelial tissues, including the cornea and retina, as well as the immune system are affected, resulting in xerophthalmia and a dysregulated immune response (95, 150). In addition, aberrant vitamin A levels in pregnant patients leads to developmental defects in the embryo (146).

### Xerophthalmia

Although xerophthalmia literally means “dry eye,” it is generally used as a term to describe the collective ocular syndromes observed in hypovitaminosis A (151), with clinical features classified by the World Health Organisation (Table 2) (152). The bulbar conjunctiva lining the globe of the eye, and not the palpebral conjunctiva lining the eyelid, is affected by xerosis, or dryness (X1A), sometimes difficult to detect (152). The superficial conjunctiva may accumulate keratin, forming foamy patches known as Bitot’s spots (X1B) (153, 154). Acute vitamin A deficiency presents with corneal pathology. Corneal xerosis (X2) is most common, as in the conjunctiva. Dryness of the corneal surface predisposes to ulceration, an ophthalmic emergency (155), and keratomalacia, characterised by diffuse keratinisation of the corneal surface, permanently impairing vision; these pathologies are categorised based on the proportion of the cornea affected (X3A/X3B). Ulcers and keratomalacia lead to permanent corneal scarring (XS). The retina is affected due to the requirement of vitamin A to produce the chromophore discussed above, essential for phototransduction. Deficiency leads to nyctalopia, or night blindness (XN) as rods are more sensitive to deficiency, and these photoreceptors underlie vision in scotopic light levels. More rarely, structural damage to rods is observed, known as xerophthalmic fundus (XF).

**TABLE 2:** World Health Organisation classification of xerophthalmia, based on clinical ophthalmological features. Shaded cells correspond to retinal pathology.

Xerophthalmia stage	WHO abbreviation
Nyctalopia	XN
Conjunctival xerosis	X1A
Bitot’s spots	X1B
Corneal xerosis	X2
Corneal ulceration/keratomalacia involving < 1/3 of the cornea	X3A
Corneal ulceration/keratomalacia involving > 1/3 of the cornea	X3B
Corneal scarring	XS
Xerophthalmic fundus	XF

Xerophthalmia is treated with large dose vitamin A supplementation, up to 200,000 IU (150, 154). Oral and intramuscular supplementation is equally effective (150). While vitamin A toxicity following supplementation is unlikely in patients with symptomatic deficiency before treatment, acutely high intake has theoretical risks associated with vitamin A’s roles in inflammation, as well as the contribution of retinoid products to Stargardt’s disease, discussed below.

### Immune dysregulation

It has been observed that in populations where vitamin A availability from food is low, infectious diseases can precipitate vitamin A deficiency by decreasing intake, decreasing absorption, and increasing excretion (93). Vitamin A deficiency has profound effects on innate immunity, including impeding normal regeneration of mucosal barriers damaged by infection, impairing mucosal inflammatory responses contributing to the synthesis of antibodies, especially IgA and by diminishing the function of neutrophils, macrophages, and natural killer cells (93). Vitamin A deficiency in viral infections, such as measles, has been shown to increase disease severity and appropriately timed supplementation during recovery has been found to reduce mortality and hasten recovery (156). The role of Vitamin A in T-cell-mediated immunity is discussed above, with reference to Treg and Th17. In addition to these effects, vitamin A deficiency has been shown to diminish Th2-mediated antibody responses and may also impair Th1 responses, whereas high dietary vitamin A may enhance Th2-mediated responses (7, 93, 157). This is thought to contribute to the increased mortality observed in vitamin A-deficient children and pregnant women.

### Hypervitaminosis A

Vitamin A deficiency is treated with supplementation, and vitamin A supplements are widely used with no adverse effects. However, acute toxicity is possible. Excessive vitamin A intake is generally associated with excessive use of vitamin A-containing supplements (158, 159). Persistently high intakes can result in overt vitamin A toxicity, described as hypervitaminosis A syndrome (160), and may exacerbate Stargardt’s disease (161).

Women of child-bearing age are cautioned not to consume high amounts of vitamin A-rich foods such as organ meats. The use of prescription retinoids is known to be associated with birth defects and thus their use by persons of reproductive age is highly regulated, and generally not recommended outside of settings where vitamin A deficiency is a public health problem (162). On the other hand, β-carotene has not been shown to be teratogenic or to produce other symptoms of toxicity and therefore it may be considered a “safe” form of vitamin A (163). Consistently high intakes of carotenoids may result in hypercarotenaemia and deposition of carotenoids in skin, causing yellowing (164), but the condition is benign (165).

## Parainflammatory degenerative retinal disease

### Stargardt’s disease

Stargardt disease (STGD) is the most common single-gene heritable disease of the retina, with three genetic bases: STGD1 caused by autosomal recessive ATP-binding cassette, sub-family A, member 4 (ABCA4) mutations (166); STGD3 caused by autosomal dominant mutations in “elongation of very long chain fatty acid protein 4” (ELOVL4) (167); STGD4 caused by autosomal dominant mutations in prominin 1 (PROM1) (168). STGD2 was attributed erroneously to a distinct genetic locus, but is in fact attributable to ELOVL4 mutations, and is now diagnosed as STGD3. STGD1 is the most common form of Stargardt’s (169), driven by a lack of functional ABCA4. Clinically, patients present with bilateral central visual loss and dyschromatopsia, due to macular atrophy (170, 171). On examination, the fundus exhibits characteristic yellow-white lesions, centred on the retina (172). Onset is usually early in life, with peaks in childhood and early adulthood; later onset is associated with better prognosis (173, 174). The disease tends to progress gradually, outward from the central macula, often over years (171, 175–177). Wide variability in disease trajectory, between patients with particular mutations as well as within individuals with similar genetics indicates dependence on both genetic and environmental factors (178, 179).

**FIGURE 4:**
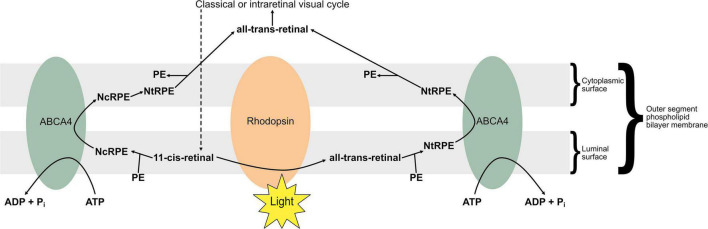
The “flipping” function of ABCA4. 11-cis retinal accumulates in darker conditions as opsins are saturated, whereas all-trans retinal is produced by photoisomerisation in lighter conditions. Retinal associates with membranal phosphatidylethanolamine (PE) to form N-cis-retinylidene-PE (NcRPE) and N-trans-retinylidene-PE (NtRPE), which ABCA4 flips across the membrane at the cost of ATP hydrolysis. NcRPE and NtRPE dissociate, and 11-cis retinal undergoes chemical isomerisation, allowing all-trans retinal to reenter the visual cycle.

ABCA4 encodes an active transporter highly expressed in the retina (180), specifically localising in the outer segments of rod and cone photoreceptors according to immunofluorescence studies (181, 182). The protein functions as a retinoid “flippase,” facilitating excretion of all-trans-retinal following light responses (183), as well as excess 11-cis-retinal which otherwise accumulates in dark conditions (184). Unlike most ATP-binding cassette proteins, ABCA4 acts as an importer, specifically of N-retinylidene-phosphatidylethanolamine (NRPE) (185), a Schiff-base conjugate of PE and retinal (186), in either an N-cis (NcRPE) or N-trans (NtRPE) conformation, associated with 11-cis and 11-trans-retinal, respectively (187). The protein functions as a retinoid “flippase” of NRPE from the luminal to the cytoplasmic side of the phospholipid bilayer that comprises the outer segment disc membrane (187), facilitating excretion of all-trans-retinal following light responses (183), as well as excess 11-cis-retinal which otherwise accumulates in dark conditions (184). All-trans retinal produced by photoisomerisation binds to PE to form NtRPE in the outer segment disc membrane, which ABCA4 “flips” towards the cytoplasm for entry into the visual cycle, to regenerate chromophore (187). 11-cis-retinal chromophore can be produced to excess in the darkness; it also binds with PE to form NcRPE in the outer segment membrane, which is similarly “flipped” towards the cytoplasm by ABCA4, where it is isomerised into NtRPE before dissociating into PE and all-trans-retinal which re-entering the visual cycle (187). This “flipping” function of ABCA4 is illustrated in Figure 4.

A lack of functional ABCA4 at the rim region of outer segment disc membranes underlies the majority of STGD1 cases (187): this can be due to loss of ATPase activity, NRPE “flipping” activity, mislocalisation of protein, or protein misfolding (182, 186, 188, 189). As a result, NRPE, therefore retinal, is not removed from disc membranes as efficiently, leading to retinoid accumulation. These retinoids may give rise to pyridinium bisretinoids including A2PE (190). A2PE undergoes hydrolysis by phospholipase D to form A2E, a fluorophore and major component of lipofuscin (187, 190, 191), which characteristically accumulates in STGD1 (183, 192). Lipofuscin and other bisretinoids like A2E have a toxic effect on the photoreceptors and are thought to underlie the STGD1 disease process (184, 189, 192–194). It is lipofuscin accumulation underlying the characteristic yellow-white lesions on fundoscopy in STGD1, giving the retina a beaten-bronze appearance (172, 195).

### Age-related macular degeneration

Age-related macular degeneration is a common cause of vision loss in the ageing population, growing in prevalence (196, 197). Patients initially experience subtle disturbances to their vision, which progress over time to permanent loss of central vision, as disease locates primarily to the central retina, or macula, as in STGD (198). AMD occurs in two distinct forms, “dry” and “wet,” with wet AMD contributing to the vast majority of severe central visual loss (199). In dry AMD, progressive loss of vision occurs due to degeneration of the choriocapillaries, outer retina, and photoreceptors (200). Outer retinal atrophy is generally observed earliest, with eventual progression to “geographic atrophy,” where the outer retina and RPE are completely atrophic (201). Wet AMD is characterised by neovascularisation, driven by immune cells recruited to the damaged macula. Cytokines underlie immune cell recruitment and neovascularisation, with vascular endothelial growth factor (VEGF) being especially involved in wet AMD (198). The earliest sign of all forms of AMD is drusen: subretinal deposits of lipids, proteins, and carbohydrates which appear as white or yellow deposits on fundoscopy, comparable to lipofuscin (198, 202).

The aetiology of AMD is complicated, with a wide range of risk factors, both modifiable (e.g., smoking) and non-modifiable (e.g., family history) (199, 203). Particular genotypes are associated with AMD, and as in STGD, ABCA4 has been specifically implicated (204, 205). Accumulation of bisretinoids such as A2E is associated with AMD as well as STGD, and administration of exogenous A2E induces ocular changes similar to AMD and STGD in animal models (206–208). However, defining a causal link between ABCA4 mutations and AMD has proven more challenging, as some researchers find no association (209, 210), perhaps due to genetic heterogeneity in the AMD phenotype, or incomplete penetrance of disease-causing alleles (211). An added complication lies in the comparable presentations of Stargardt’s and AMD, albeit usually in younger and older patients, respectively, with consensus favouring the role of ABCA4 as limited to Stargardt’s and related retinal dystrophies and mimicking, rather than causing AMD (211, 212). ABCA4 is nevertheless included in the EYE-RISK genotype assay for AMD, as retinal dystrophies are an important differential diagnosis in patients presenting with macular pathology (212).

### Retinoic acid and parainflammation in the retina

Parainflammation, defined as an adaptive tissue response to noxious stress, between basal and inflammatory states, has been proposed as a mechanism for the pathogenesis of degenerative retinal disease (213, 214). Furthermore, immunosenescence (altered immune functions during ageing) is thought to play an important role in AMD, which could be considered a systemic immunologic disease, with local expression in the retina, due to the decline of the ocular down-regulatory immune environment (30). Oxidative stress in the retina increases with age, with reactive oxygen species (ROS) affecting metabolic processes (215, 216). ROS are produced physiologically, such as due to lipofuscin accumulation or due to environmental influences, including ultraviolet (UV) radiation from sun exposure, or other modifiable AMD risk factors such as smoking (217). Inflammatory processes are also thought to contribute, particularly from tissue-resident immune cells and supporting cells, such as Müller glial cells (216). Macrophages appear to be particularly involved in AMD, with preferential differentiation of the pro-inflammatory M1 phenotype, as opposed to the M2 phenotype associated with regular ageing (218, 219). As the retina is exposed to physiological stress, immune activation may exacerbate cellular damage, leading to loss of function (214). In Stargardt disease, the A2E vitamin A cycle byproducts form abnormally in the young retina, whereas in AMD, the byproducts form, to a similar degree, in the aged retina and it is hypothesised that the difference in the two diseases is explained by the age of the eye in which the vitamin A cycle byproducts (208). Determining the effects of dietary vitamin A on parainflammation in STGD1 and AMD is complex as it requires consideration of the pleotrophic effects of RA-signalling and its targets (in the eye, gut or other immunological sites), the age of the eye, the extent of local retinal disease, as well systemic immunological factors. Whereas RA is known to act directly on macrophages at mucosal and other immunological sites to modulate inflammation (93, 220), the effect of RA on local immune antigen cells residing in damaged RPE may well be pro-inflammatory, as a result of aberrant Vitamin A processing. On the other hand, the parainflammatory response to deposition of toxic metabolic byproducts could potentially be modulated by RA-dependent iTreg generation in the microbiome environment. Dietary vitamin A is likely to influence local retinal parainflammatory processes through the effects of RA signalling on the microbiome environment, systemic T cell homeostasis and resident retinal immune cells but whether the overall effect of RA is beneficial or detrimental to retinal disease is yet to be determined.

## Clinical applications and directions for research

No curative treatments exist for STGD or AMD. Current research may lead to improved options relating to pharmacology, stem cells, laser treatment, and implantable intraocular lens telescopes (221). The quality of evidence for potential treatments is variable (221). Nutritional and retinoid- and carotenoid-related approaches are explored below, with human evidence summarised in Tables 3, 4.

**TABLE 3:** Human-based experimental studies relating to vitamin A treatment for STGD, with PICO characteristics summarised.

Citation	Participants	Interventions	Comparisons	Outcomes
Sofi et al. (225)	24 STGD patients	High vitamin A intake	Low vitamin A intake	Visual acuity
Kong et al. (226)	259 STGD1 patients	Vitamin A supplementation	No supplementation	Visual acuity
National Institutes of Health (232)	40 healthy adults	C20-D_3_-vitamin A	–	Safety evaluation
National Institutes of Health (233)	140 STGD1 patients aged 8–70 years	C20-D_3_-vitamin A	Placebo	Safety and tolerability; STGD lesion size; visual acuity; ocular assessment; pharmacokinetic profile

STGD, Stargardt’s disease; PICO, participants, interventions, comparisons, outcomes; C20-D_3_-vitamin A, carbon-20 deuterium-enriched vitamin A.

**TABLE 4:** Human-based studies relating to vitamin A in AMD, with PICO characteristics summarised.

Citation	Participants	Interventions	Comparisons	Outcomes
Age-Related Eye Disease Study Research Group (241)	3640 AREDS patients aged 55–80 years	Antioxidant supplement including β-carotene; zinc/copper supplement; antioxidant and zinc supplement	Placebo	Progression to advanced AMD; visual acuity loss
Owlsey et al. (237)	104 adults within AREDS steps 1–9	High dose retinol	Placebo	Dark adaptation
Age-Related Eye Disease Study Research Group (238)	4,519 AREDS patients aged 60–80 years	Dietary lutein/zeaxanthin intake	–	Neovascular AMD; geographic atrophy; large/extensive drusen
Age-Related Eye Disease Study 2 Research Group (239)	4,203 AREDS patients aged 50–85 years	Lutein/zeaxanthin supplement; docosahexaenoic acid/eicosapentaenoic acid supplement; lutein/zeaxanthin/docosahexaenoic acid/eicosapentaenoic acid supplement	Placebo	Development of advanced AMD
Munch et al. (243)	848 Inter99 patients aged 30–60 years	Vitamin A intake	–	Macular drusen
Agrón et al. (242)	4504 AREDS patients without AMD at baseline	Vitamin A and carotenoid intake	–	Progression to advanced AMD
Chew et al. (244)	3882 AREDS patients	β-Carotene supplement	No β-carotene supplement	Self-reported late AMD

AMD, age-related macular degeneration; AREDS, age-related eye disease study grading system; PICO, participants, interventions, comparisons, outcomes.

### Stargardt’s disease

Given that the pathophysiology of STGD depends on accumulation of retinoids in photoreceptor outer segments, various research groups have hypothesised that vitamin A levels ought to correlate negatively with outcome in patients lacking functional ABCA4. A mouse-model for STGD1 has been developed, by functionally knocking out the ABCA4 gene (222). Although genetically and phenotypically similar to human STGD1 patients, the validity of this model has been questioned as ABCA4-knockout mice are healthy unless exposed to very bright light (223). The relevance of ABCA4-knockout models to other STGD genotypes, with pathogenic mutations outside ABCA4, is also questionable (169). In ABCA4-knockout mice, no association is apparent between vitamin A supplementation and retinal morphology and structure (161, 224), although greater lipofuscin accumulation following vitamin A supplementation was noted in one study (161). Studies in humans are not much more conclusive than results in animal models. A small cross-sectional study of 24 patients assessing vitamin A intake with diet questionnaires found low vitamin A intake associated with better visual acuity (225). However, a larger prospective cohort study (*n* = 259) found vitamin A supplementation had no effect on visual acuity at baseline, or over 12 months (226).

Another potential strategy is based on deuterium-enriched vitamin A (C20-D_3_-vitamin A), which reduces vitamin A dimerisation through competitive inhibition, thereby slowing A2E biosynthesis (227). C20-D_3_-vitamin A has the added benefit of reacting normally throughout the visual cycle, therefore not impinging upon chromophore generation which is required for visual transduction (228). In ABCA4-knockout mice, C20-D_3_-vitamin A has exhibited conflicting results: one study finding greater electroretinogram responses with C20-D_3_-vitamin A supplement relative to non-deuterated vitamin A supplement control (229); one study finding no differences between similar groups, though vitamin A dimerisation was inhibited (230); another study finding that C20-D_3_-vitamin A inhibited prodromal pathological features (231). Following a successful phase 1 clinical trial assessing the safety and pharmacokinetics of C20-D_3_-vitamin A (232), a larger phase 2 clinical trial assessing deuterated vitamin A’s long-term safety and tolerability is currently recruiting (233).

Carotenoid supplementation, which provides a source of retinoids, has been suggested as a potential nutritional approach to improving STGD outcomes by protecting the macula (221), due to observed lower carotenoid serum levels in STGD patients compared to controls (234). While carotenoid supplementation has a positive effect on visual performance in ABCA4-knockabout mice (235), no nutritional trials have been conducted as of yet (219). Although there is conflicting evidence regarding dietary vitamin A and STGD outcomes (225, 226), laboratory evidence of vitamin A supplementation driving greater lipofuscin accumulation (161), albeit without significant effects on retinal function (57, 224) has led to recommendations for STGD patients to avoid retinoid supplements (221). Whether or not potential differential effects of dietary carotenoids and retinoids are related to differences in the gut microbiome of STGD patients is not clear; if demonstrated, this would represent another avenue for experimental enquiry.

### Age-related macular degeneration

Investigating the effects of nutritional supplements in AMD is complicated, as although serum levels of retinoids or carotenoids may increase quickly, changes in the macular may occur over months, and effects on visual function could take even longer, perhaps years, to manifest (236). Nevertheless, retinoid and carotenoid deficiency are implicated in the progression of AMD (237, 238), and evidence from large randomised controlled trials of nutritional supplementation in ophthalmology patients, AREDS1 and AREDS2 (239, 240), suggests that sufficient levels of vitamin A or β-carotene was associated with decreased risk of late AMD, neovascular AMD, geographic atrophy, and large drusen accumulations (239, 241, 242). This agrees with the results of a meta-analysis of six longitudinal cohort studies examining the impact of carotenoids on AMD prevalence (229). However, one study found a greater risk of drusen in patients with high vitamin A intake, perhaps suggesting that supplementation increases the risk in patients with very early stage disease (243). In terms of the specific nutrient to supplement, zeaxanthin is preferred to β-carotene, as the latter tends to compete with other supplemented nutrients for absorption (221). Lutein and zeaxanthin supplementation appears to associate with better outcomes in late AMD than β-carotene (244).

It remains an open question as to how nutrition can be optimised to improve outcomes in STGD1 and AMD. While retinoids are critical for vision, as explained above, it may be the case that ingesting too much accelerates the pathogenesis of STGD1 (161, 225). Retinoids and carotenoids are often deficient in AMD, carotenoid supplementation appears to be beneficial (229, 244), and vitamin A supplementation inhibits IL-17 and ROR expression in atherosclerosis, which features comparable macrophage-driven pathophysiology to AMD (245). However, certain data suggest that there may be a risk of worsening outcomes in patients with early-stage disease (243). The role of the gut microbiome in affecting treatment options remains unclear, and no attempts have been made to leverage observed differences in STGD and AMD patients in terms of clinical trials. Further research is required to (a) determine the optimal amount of dietary and retinal vitamin A to maintain normal physiology while slowing, or at least without accelerating disease processes; (b) explore whether different sources of vitamin A have different effects on Stargardt’s, for instance comparing vitamin A retinoids and provitamin carotenoids; (c) differentiate between different disease phenotypes and disease phases, and associate differential effects of vitamin A and associated therapies on their pathophysiology accordingly; and (d) investigate the influence of the gut microbiome on STGD and AMD, and develop effective therapeutics based on observed differences.

## Conclusion

The group of fat-soluble compounds referred to as vitamin A is essential to humans, and has particularly significant roles in vision and T-cell mediated inflammation, as well as in a wide range of other physiological systems. Its importance is illustrated by the deficiency syndrome xerophthalmia, particularly affecting the eyes. In addition to maintaining the cornea, vitamin A is critical for the function of the retina, as it provides the raw material to produce the chromophore, 11-cis-retinal. Photoreceptor cells require the chromophore to transduce light energy for vision, and regenerate chromophore in collaboration with the RPE and Müller cells through a series of reactions known as the visual cycle. Bisretinoid byproducts of photoisomerisation and the visual cycle include A2E and lipofuscin, which can accumulate with associated toxicity to rods and cones. Vitamin A also has a critical signalling role in the immune system, with particular effects on T cell differentiation across the body, including in the intestine, in close association with the gut microbiome. Vitamin A deficiency is associated with susceptibility to infections, causing significant morbidity and mortality worldwide, as well as predisposition to autoimmunity and hypersensitivity.

In STGD1, a lack of a membrane exchange protein leads to accumulation of bisretinoids, ultimately derived from vitamin A, which culminates in blindness progressing from the central visual field as the macula is affected first. AMD similarly affects the macula, and features drusen comparable to the lipofuscin deposits of STGD, but instead primarily containing lipids and other waste products. Various retinoid and carotenoid-based approaches to treating STGD and AMD have been posited. In particular, zeaxanthin supplementation is well-evidenced as beneficial for AMD patients, while vitamin A supplementation may worsen STGD1 outcomes. However, deuterium-enriched vitamin A could target STGD1 with high specificity, and is currently in the clinical trial stage. No accepted or trialled therapies have leveraged the altered gut microbiome in STGD or AMD, and this may represent an interesting area for further research. As no curative treatments exist for STGD or dry AMD, novel approaches are necessary to improve outcomes for patients, and optimising nutrition represents an important facet of management.

## Author contributions

AJT and RMG undertook editing. AJT and ACR produced figures. AJT organised references. All authors contributed to research and writing and approved the final draft for submission.

## Conflict of Interest

The authors declare that the research was conducted in the absence of any commercial or financial relationships that could be construed as a potential conflict of interest.

## Publisher’s Note

All claims expressed in this article are solely those of the authors and do not necessarily represent those of their affiliated organizations, or those of the publisher, the editors and the reviewers. Any product that may be evaluated in this article, or claim that may be made by its manufacturer, is not guaranteed or endorsed by the publisher.
